# Supplementary material for: Evaluation of In2Care mosquito stations for suppression of the Australian backyard mosquito, Aedes notoscriptus (Diptera: Culicidae)
Source: J Med Entomol. 2023 Aug 3;60(5):1061–72. doi: 10.1093/jme/tjad099 (PMC10496431; doi:10.1093/jme/tjad099)
Supplement: tjad099_suppl_Supplementary_Material [file tjad099_suppl_supplementary_material.docx]

**Supplementary material**

**
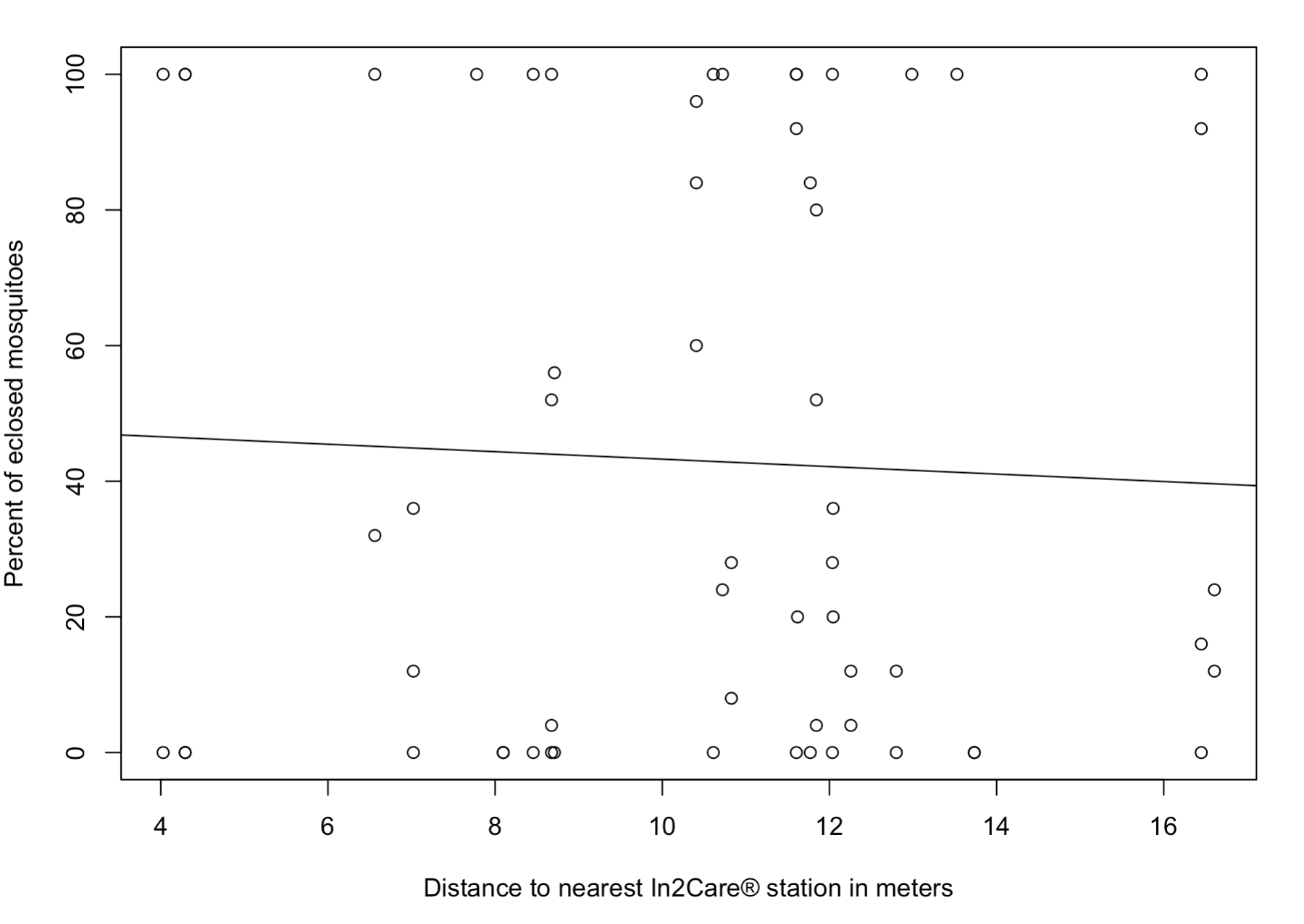
**

**Figure S1 Scatter plot of a Mantel test between the distance of water samples collected from ovitraps in the treatment site to the nearest In2Care® station (x-axis) and the percent of eclosed mosquitoes from those water samples (y-axis).** Line describes linear regression fit.
